# Promoting factors and barriers to participation in working life for people with spinal cord injury

**DOI:** 10.1186/s12995-020-00288-7

**Published:** 2020-12-17

**Authors:** Christian Sturm, Andrea Bökel, Christoph Korallus, Veronika Geng, Yorck B. Kalke, Rainer Abel, Ines Kurze, Christoph M. Gutenbrunner

**Affiliations:** 1 Department of Rehabilitation Medicine, Hannover Medical School, Carl-Neuberg Str. 1, 30625 Hanover, Germany; 2 Manfred-Sauer-Foundation, Lobbach, Germany; 3 RKU – University and Rehabilitation Clinics Ulm, Ulm, Germany; 4 SCI Unit, Klinikum Bayreuth GmbH, Bayreuth, Germany; 5 Department for Paraplegia and Neuro-Urology, Centre of Spinal Cord Injuries and Diseases, Bad Berka, Germany

**Keywords:** Disability studies, Employment, Social security, Spinal cord injuries, Unemployment

## Abstract

**Background:**

It is still difficult for people with physical impairments to be and remain equally integrated into the labour market. For this reason, the question of occupational activity has explicitly been examined by the German Spinal Cord Injury Survey (GerSCI) in order to identify barriers and facilitators for labour market participation.

**Methods:**

Cross-sectional explorative observational study. The GerSCI survey is the German part of the International Spinal Cord Injury Survey (InSCI). Using survey data from persons recruited at eight specialised SCI-centres in Germany. Participants: 1.479 persons with Spinal Cord Injury (SCI) aged 18 years and older.

**Results:**

In a self-disclosure questionnaire, persons with SCI show themselves as a professionally well-educated and highly motivated group with most of them aiming at gainful employment and considering themselves fit for work. Many changeable and non-changeable factors have been found, which showed a high correlation with the return to work after acquired SCI.

**Conclusion:**

Education and pain belong to the most critical factors and thereby possible approaches to increase the level of employment, which is essential and highly relevant not only for earning money but also for self-confidence and social integration. SCI has many dimensions in itself; support also should be multidimensional. Study results might help to improve participation.

## Background

Many workers leave the labour market permanently due to a health problem or disability. Too few people with reduced working capacity manage to remain in employment in member countries of the Organisation for Economic Co-operation and Development (OECD) [1]. The financial situation of those concerned has been improved in many countries of the OECD through existing social security systems. Social participation is nevertheless inadequate, and the financial burdens at both individual and societal level are high. This issue is one of the biggest social and labour market challenges for policymakers [1].

In Germany, the support for participation in working life of people with disabilities is anchored in the Social Code Book IX (§ 166 SGB IX) in the so-called inclusion agreement. This agreement regulates the cooperation between the employee representatives, in particular the representative body for persons with severe disability, and the employer. They have to agree on objectives for improving the integration of persons with disability in the respective company. This agreement legally obliges the employer to conduct such consultations and negotiations [2]. While this has already led to some improvements in recent years, people with disability are still underrepresented in gainful activity. The unemployment rate in Germany for people with severe disabilities (11.2%) is more than twice as high as in the general labour market (5.2%). Persons with disabilities experience difficulties to enter the labour market, which is being reflected in the fact that it takes them 100 days longer on average to find suitable employment [3]. Data availability of studies on the subjective life situation of people with SCI had been analysed before the implementation of this study, revealing a very heterogeneous situation between the different countries. In the case of Germany, no conclusive study on this subject was found in the last years [4]. From a clinical point of view, even while up to 75% of the patients were deemed to be suitable after in-patient rehabilitation, only 30% could be reintegrated into working life in Germany [5].

A special feature in Germany is that the cost units are different. If an accident at work has led to SCI, the German Social Accident Insurance (DGUV) pays the therapy. Otherwise mostly the health insurance will pay for the clinical treatment. Rehabilitation and professional reintegration measures then usually are financed after primary care by the German Pension Insurance (DRV).

The specific aims of this study were 1) to describe the prevalence of labour market participation in the German study population and 2) to analyse determinants of labour market participation across relevant subpopulations based on demographic data, social and health-related factors, and SCI characteristics.

## Methods

In the case of acquired Spinal Cord Injury, people often experience this as a hard cut also concerning training or working life. In order to study this in more detail, data from the German Spinal Cord Injury Survey (GerSCI) were used. The GerSCI survey is the German part of the International Spinal Cord Injury Survey (InSCI). InSCI is a multinational community survey based on the International Classification of Functioning, Disability and Health Core Sets for SCI and involves 22 countries from all six WHO regions [6]. First results in international comparison showed that the successful implementation of the InSCI survey enables to compare the lived experience of persons with SCI across the globe [7]. GerSCI was implemented from March until December 2017 for people with SCI treated at eight of 27 specialised SCI rehabilitation centres. The study was conducted by the Department of Rehabilitation Medicine at Hannover Medical School (MHH). It was approved by the Ethics committee of the MHH, following the Helsinki Declaration of 1975 (No. 7374), as well as the Commissioner for Data Protection at MHH.

### Inclusion criteria:

- presence of acquired SCI (traumatic or non-traumatic)
- Age ≥ 18 years
- Completed post-acute rehabilitation: 12 months after the onset of the spinal cord lesion
- Current place of residence: Germany, language competence: German

### Exclusion criteria:

- Congenital SCI or neurodegenerative diseases

From the database of the eight participating specialised German SCI centres, 5,598 potential participants were identified. They then received an invitation letter containing a questionnaire, which they could answer either electronically or in paper form. At the end of the recruitment period and after checking for exclusion criteria, 1,479 questionnaires were taken into account for subsequent data analysis [8].

### Survey instruments

The underlying InSCI data model is based on a similar approach as the WHO World Health Survey, which employed key health components as the basis for the questionnaire design. For the InSCI data model, the International Classification of Functioning, Disability and Health (ICF) served as a guide in selecting the most appropriate categories, to fully describe the life experiences of people with SCI, i.e. to assess what is most important for the affected person [6].

Due to the study being designed as an international research project, and to ensure the comparability of the data of all participating countries, the questionnaire had been developed in English by the InSCI study coordination team and then later translated to German. The questionnaire consisted partly of items of validated instruments, partly of questions that had been agreed upon by consensus [9].

### Additional questions, additional answer options, national modules

Each participating country had the option to include questions with national relevance in the questionnaire. The following changes were made that are relevant for this study evaluation:

The question concerning the level of education was split into two separate, more detailed questions. The question of whether the pain impeded someone from pursuing a professional activity was added.

In respect to the question on vocational re-integration measures, the answer option: “vocational re-integration measures did not exist at that time” was added [8].

### Covariates

Sociodemographic data and lesion characteristics (age, gender, lesion characteristics, etiology), as well as education, were used to analyse associations.

### Statistical analysis

Sociodemographic data and SCI characteristics are presented as percentages or means with a standard deviation (SD). The key focus of this study was the labour market participation of people with SCI in Germany. The labour market participation frequencies are presented as sum totals in addition to percentages. Only data from participants with less than 30% missing values were included in the statistical analysis.

To analyse the determinants of labour market participation and identify disadvantaged groups, covariates associated with participation were assessed using multivariable logistic regression and estimated odds ratios (OR) with a 95% CI. To assess chances and risks logistic regression in order to determine probabilities for important factors of becoming employed or not was calculated. Values that were conspicuous in the analysis before were chosen.

Results were considered statistically significant if p-values were less than 0.05. Statistical analyses were performed with SPSS 26.0, IBM.

## Results

### Epidemiology

The mean age of the 1,479 participants was 55.3 years (SD = 14.6), 73% of them were male. About half of the participants stated tetraplegia (10.4% complete, 38.3% incomplete), and half paraplegia (23.8% complete, 27.5% incomplete). On average, the participants had already had SCI for 14 years at the time of the survey (range 1–4 years up to over 30 years).

73.6% of the participants belonged to the working-age population (*n* = 1,088) between 18 and 65 years of age. Of these, 42.5% were actually in gainful employment at the time of the survey. However, there was a gender gap in employment: 43.9% of men were in work, but only 38.9% of women.

Trauma was the most frequent cause of SCI in 77.8% of the working-age respondents. In all other cases (22.2%) a non-traumatic pathology, for instance, the vascular disease was the cause. Predominantly, the professional activity consisted of an employment relationship. At the time of the survey, 2.1% declared work in their household as their occupation, 2.4% were students, and 5.2% were registered as unemployed (Table 1).

**Table 1:** Breakdown of gainful activities of the survey respondents

Current employment situation										
	Work for wage/salary	Work for wage/salary, but sick	Self-employed	Non-paid family-member	Housewife / House-husband	Student	Unemployed	Pension due to state of health	Old-age pension	Other employment
**Male (%)**	37,1	2,7	5,6	0,5	1,0	2,2	5,2	41,7	1,8	2,1
**Female (%)**	35,4	3,2	2,8	0,0	4,9	2,8	5,3	42,1	2,8	0,8
**Total (%)**	36,7	2,8	4,8	0,4	2,1	2,4	5,2	41,8	2,1	1,7
**Total (n)**	386	30	51	4	22	25	55	440	22	18

Sixty-five percent of respondents in working-age received financial support based on their state of health, such as benefits for a reduction in earning capacity. Forty-one percent received early retirement benefits due to the state of their health.

The average working time per week of people with a paid job was 28.4 h (SD = 11.9). Analysis of the group distribution demonstrated that about half of the respondents (51.3%) worked up to 30 h a week, whereas the other half worked more hours (48.7%). However, 24% of the respondents indicated that they would like to reduce the number of hours per week.

The majority of people in gainful employment felt that their work was adequately valued (fully agree: 38.9%, agree: 48.0%), but a proportion of 24.6% felt that they were not adequately remunerated for their work. 11.3% of respondents reported that they had difficulties meeting the demands of the job (major or extreme problems).

**Fig. 1:**
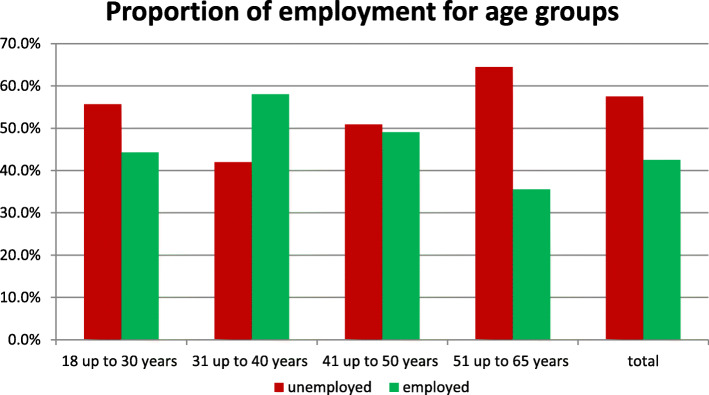
Employment rate in different age groups

Age structure: within the age group 18-30y 44.3% reported a paid job, in the age group 31-40y the share of those in gainful employment was highest at 58.0%, falling to 49.1% in the age group 41-50y and reaching the lowest figure of 35.6% in the age group 51-65y (Fig. 1).

Technical aids**:** 34.2% of the gainfully employed respondents stated that they did not need adapted technical aids. However, more than one fourth (26%) reported they had slightly limited (10.3%), limited (9.0%) or no access (6.7%) to aids they needed.

### Unemployment

Of all working-age respondents, 57.5% were not in gainful employment at the time of the survey.

When considering the characteristics of the SCI, persons with tetraplegia (62.5%) were more likely to be without paid employment than respondents with paraplegia (54.8%) (OR = 1.5, CI = [1.17—1.91]. Likewise, persons with disease-related paraplegia were more likely to be unemployed (64.6%) than people with injury-related (55.2%) (OR = 1.5; CI = [1.10–2.00]. The data also demonstrate that respondents without paid employment were on average about four years older (*p* < 0.02; d = 0.32), and were also on average five years older at the onset of the illness (*p* < 0.01; d = 0.35).

The inability to sit without support is correlated with a strikingly high rate of unemployment of 71.9% (*p* < 0.001). In contrast, whether the lesion was complete or incomplete seems to have little effect, as the ratio of employment to unemployment was almost equal (complete 42.3% vs 57.7% and incomplete 42.8% vs 57.2%).

Fifty-nine point five percent of the respondents without paid employment stated the desire to work again. Of these, 71.4% felt that they were quite capable of pursuing a profession.

Fifty point nine percent of the people between 18 and 65y felt that they were not in a position to pursue a paid activity. One-fourth of the respondents believed they could work between one and 11 h per week, while another quarter thought they could work between 12 and more than 20 h. Even in the subgroup of unemployed people with the desire to work, the rate of those considering themselves incapable of working still amounted to 28.6%.

Participants were also surveyed about the causes that hinder their gainful employment. The most common answer (64%) was that the state of health and disability was the main cause of unemployment. Often (36%) no matching job with a suitable profile could be found, and 12% of the participants also stated that they did not even know how to look for a suitable job.

More than one fifth (22.7%) reported workspace environments which were not barrier-free and therefore not accessible without great effort. For 15.5% the way to work posed a barrier preventing them from employment. In 11% of the cases, lack of adapted aids was given as a reason for limiting the ability to work.

**Fig. 2:**
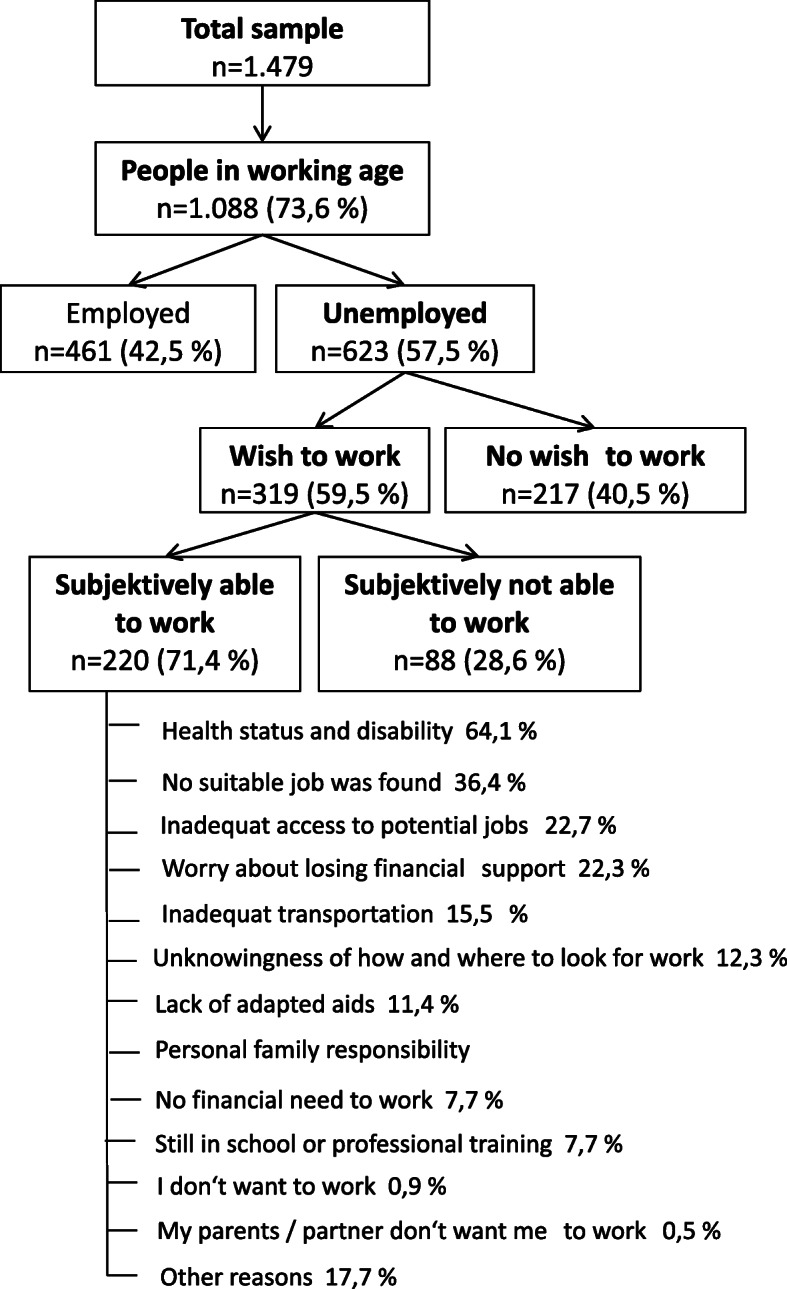
Flow chart of the causes of unemployment

Furthermore, financial aspects played a role: Almost 8% responded they had no financial need for a job and more than 22% expressed worries about the loss of financial support after resuming work (Fig. 2).

**Fig. 3:**
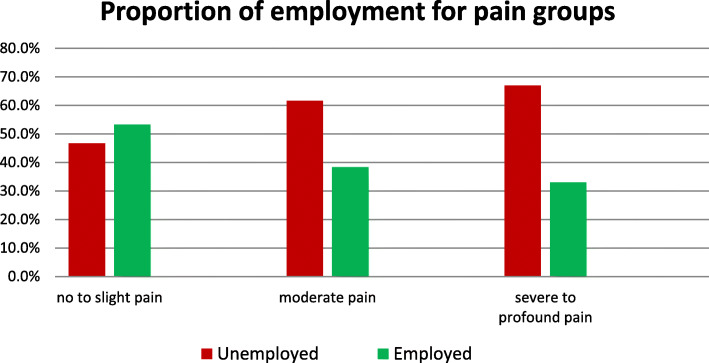
Pain grouped with or without gainful employment

From a medical point of view, the impairment caused by pain is an essential consideration. Since 55.5% of the unemployed stated that pain in everyday life prevents them from working, this seems to be a particularly important point. Even among those who have a job, 13% stated that they are hindered in their work by pain. Unemployment rates vary from 38.7%, for respondents who rated the pain experienced with the lowest value of 0 to 88.5% for those rating highest pain level at 10. While 53.3% in the group with no or only a little pain report to be gainfully employed, this share declines to 38.3% in the group with moderate pain, and further drops to 33.0% in the group with severe pain (Fig. 3).

### Education

Sixty-eight point eight percent of the participants have completed professional training, and another 19.9% have graduated from university or similar. Only 6.0% stated that they neither had any degree nor were in continuing education.

Overall, a clear trend could be derived from the data: the higher the respondents' level of education, the higher their employment rate. Divided according to this, it becomes evident that employment level continuously increases from 14.1% at the lowest level without a degree to 76.9% with the highest level as PhD or with habilitation (Table 2).

**Table 2:** Ratio employed to unemployed for each professional education level

**Highest professional educational level**	**Gainful employment**		**Total number (n)**
	**No (%)**	**Yes (%)**	
No professional educational	85,9	14,1	64
In current education	38,5	61,5	26
Vocational and operational training	65,6	34,4	468
Vocational school education	47,3	52,7	110
Technical college, master craftsman school, vocational or technical academy	52,9	47,1	157
Bachelor degree	45,5	54,5	33
Diploma, Master’s, Magister or State Examination completed at (technical) college or university	36,5	63,5	167
PhD, habilitation	23,1	76,9	13
Other degree	93,3	6,7	15
No values	68,7	31,1	16
Total	57,2	42,8	1069

Measures in the health care system for re-integration into the labour market were also surveyed to assess supporting factors. With approximately 60%, the majority of those surveyed did not participate in any such support measures. However, the current employment status was an important factor in this: While 62.0% of the participants in gainful employment had taken part in a vocational re-integration measure, only 38.0% of the unemployed group had taken advantage of such support measures.

### Associated factors to employment incl. odds ratios

Logistic regression showed a significant model (Chi-square (6) = 50.663, *p* < 0.001, *n* = 1.088). It also shows that if age increases by one unit, the relative probability that a person is employed will decrease by 2.7% (0.973–1 = -0.027). Individuals with paraplegia have a 1.39 times higher probability of getting employed than persons with tetraplegia. Appears to be important if you are not able to sit without any help, your chance for being employed is only half of which with the ability to sit (*p* < 0.002) (Table 3).

**Table 3:** Logistic regression for sociodemographic and SCI-characteristic factors associated with the employment status (*n* = 1.088); *p* < 0.05 are marked bold

		(Chi-square(6) = 50.663, *p* < 0.001, *n* = 1.088) Nagelkerkes *R*^*2*^ = 0.065	
		OR (95%-CI)	*p*-values
Gender	men vs. women	1.274 (0.944 – 1.719)	0.113
Age		0.973 (0.962 – 0.962)	** < 0.001**
Lesion height	Paraplegia vs. Tetraplegia	1.390 (1.057 – 1.829)	**0.019**
Completeness of lesion	complete lesion vs. incomplete lesion	0.890 (0.669 – 1.185)	0.425
SCI cause	traumatic vs. non-traumatic	1.261 (0.904 – 1.760)	0.172
Abel to sit	No vs. Yes	0.545 (0.370 – 0.801)	**0.002**

For some important changeable factors, a regression analysis was performed in order to understand the meaning for chances. There is a high level of significance in nearly every chosen factor.

The probability of getting a job increases with the level of vocational and operational training. For persons with professional training, it is 2.99 times higher than for those without. The probability of gaining employment with a bachelor degree is 7.69 times, with a PhD 26.4 times higher than without vocational training.

Also, pain is a very important factor. Moderate or severe pain reduces the probability to get employed under 50% compared to participants without or low pain.

If the interviewed persons participated in vocational re-integration, their probability rises 4.4 times to get a job (Table 4).

**Table 4:** Logistic regression for professional training and pain factors associated with the employment status (*n* = 1.034); *p* < 0.05 are marked bold

		(Chi-square(10) = 225.55, *p* < 0.001, *n* = 1,034) Nagelkerkes *R*^*2*^ = 0.27	
		OR (95%-CI)	*p*-values
**Professional qualification**	In current education vs no professional qualification	13.517 (4.438 – 41.166)	** < 0,001**
	Vocational and operational training vs. no professional qualification	2.991 (1.380 – 6.481)	**0.005**
	Vocational school education vs. no professional qualification	5.474 (2.338 – 12.815)	** < 0,001**
	Technical college, master craftsman school, vocational or technical academy vs. no professional qualification	4.314 (1.900 – 9.807)	** < 0,001**
	Bachelor degree vs. no professional qualification	7.692 (2.667 – 22.184)	** < 0,001**
	Diploma, Master's, Magister or State Examination completed at (technical) college or university vs. no professional qualification	12.116 (5.299 – 27.702)	** < 0,001**
	PhD, habilitation vs. no professional qualification	26.421 (5.755 – 121.295)	** < 0,001**
**Pain**	Medium vs. low pain	0.477 (0.340 – 0.669)	** < 0,001**
	Strong vs. low pain	0.436 (0.310 – 0.613)	** < 0,001**
**Participation in vocational reintegration**	yes vs. no	4.434 (3.311 – 5.939)	** < 0,001**

## Discussion

In the detailed analysis of these extensive data on various parameters of the lives of people with SCI, it becomes apparent that there are in part, considerable differences between the analyzed factors. In general, the design of this study only allows correlations even if a direct causal relationship can be assumed. Logistic regression additionally helps to illustrate the relationship between the values and factors better.

The inability to sit without help was found to be a particularly striking factor in connection to the unemployment rate, which is perhaps of little surprise as being able to sit is an essential factor concerning the real working world. In an analysis from Canada regarding the physical demand attributes of 181 different occupations, it was identified that 58% of these occupations required sitting [10].

Whether an existing lesion was complete or incomplete, however, did not have a statistically significant influence in our results, which is unexpected given the fact that research in other countries had shown the severity of SCI as a negative predictor. Also, other factors like older age, being female [11] and ethnicity [12] were shown to be an unchangeable negative predictor for a return to working life.

Regarding some factors, such as the cause of SCI, various confounding aspects probably play a role. A possible cause would be being younger and more active in sports results in a higher risk for accidental injury—for example in extreme sports—explaining the higher rates of occupational activity in people with traumatic causes for SCI: younger, as well as highly motivated people probably easier return to work and—if necessary – are easier to re-train. On the other hand, persons with acquired lesions caused by non-traumatic diseases such as vascular pathologies are on average older. They probably suffer from co-morbidities, such as poorly adjusted diabetes, or heart disease, thereby giving a possible explanation for the poorer values of re-integration into working life. But such considerations cannot be clarified on the basis of the available data of this study. In addition, there are studies which found differing results with the proportion of workers with non-traumatic cause for SCI being slightly higher than with traumatic cause [13].

There is also the trend that young well-educated persons have the highest employment figures. We do not know why this does not apply to such an extent to very young people of working age. Maybe for those under 30 years of age not everyone has yet completed their training. The data showed the highest employment rate among 31 to 40-year-olds, at 58%. These results are consistent with a study from Switzerland, where the highest level of employment was at the age of 40 [13].

The high impact of the level of education shown by our data also fits in well with a high level of education that is clearly associated with a higher level of employment. Various studies have confirmed the high correlation of education to participation and working life [11].

The conclusion that an academic education enables to choose from more professions that can also be pursued with SCI seems likely, possibly because the proportion of physical work is lower on average than in some manual occupations. Conversely, this could mean that further vocational education and training for people with acquired SCI would result in higher employment rates among this group. Based on their analyses of the practised profession before and after acquired SCI, Schwegler et al. advised that future return-to-work strategies should not primarily target the clerical sector with its diminishing job opportunities. It is supposed to be better to promote vocational re-training towards jobs requiring higher education and assistive technology [14]. Another study analysed the skills for typical jobs after SCI: the participants' occupations predominantly required verbal abilities, complex problem-solving skills and were characterised by conventional work tasks, and social relationships [15]. The authors developed and tested a special tool based on databases for finding a suitable job for unemployed people with SCI and found that this seems to be helpful [16].

The data in our study indicates that vocational re-integration measures are of importance: Of those who had returned to work, 62.0% had participated in a vocational re-integration measure. However, it must be critically questioned whether younger age and a higher overall state of health and motivation resulting in higher chances of employment could also lead to receiving more support measures. On the other hand, some people in Germany are sent into retirement, because effort of re-integration does not seem justified.

A meta-analysis found that the rate of employment of persons with SCI in different countries ranges from 11.5% to 74% [17]. In comparison to the results of similar studies from Switzerland (employment rate 53.4%) [13], Taiwan (employment rate of 30.3%) [18], Canada (employment rate 38%) [10], Italy (employment rate 42.1%) [19] and the Netherlands (employment rate 60%) [20], the percentage of persons with SCI employed in Germany (employment rate 42.5%) is in the middle range. It should be noted, however, that some of these studies were conducted solely on patients with traumatic causes of SCI.

So, if 59.5% of the unemployed have the desire to return to work, and 71.4% of these persons realistically see themselves capable of pursuing a profession at least part-time, what are the reasons for this discrepancy between aspiration and reality?

In order to derive concrete options for action, it is sensible to sort the influencing factors into changeable and non-changeable ones.

Impairment factors such as lesion height, age, gender or the occupation before SCI cannot be influenced. However, the spectrum of possible new professional activities can be influenced by professional training measures. Further, frequent consequences of SCI such as depression, bladder dysfunction, spasticity and other similar physical factors which are also important both in rehabilitation and workability can also be positively influenced [21]. For example, returning to work was positively correlated to wheelchair capacity at discharge from the rehabilitation department [22].

Results from the Swiss SCI Cohort Study presented that pain (in the past week) was reported by 68.9% and chronic pain by 73.5% [23]. Optimising pain management thus seems necessary, as many patients still report that pain prevents them from going to work, and the reported pain scores in this study are high overall. The probability to get employed doubles if the pain level is low. A recent study confirmed that the presence of significant pain and the use of either prescription painkillers or non-prescription opioids could have a significant negative impact on both the likelihood and the quality of employment [24]. Pain in this clinical field is a hard challenge. Especially, because several types of pain typically present following SCI with central neuropathic pain being a frequent and difficult to manage occurrence [25]. Patients often can only choose between a good reduction of pain and having many side-effects like dizziness (and are not able to work because of the side-effect) or bear up against a moderate level of pain and reduce side-effects.

Factors such as local public transport, conversion of cars, accessibility of buildings and accessibility of workplaces are complex and partly a costly matter but can nevertheless be influenced well. Studies from Switzerland and Italy also point to the high correlation between mobility and employment [19, 26]. Concerning these factors, political and state measures overlap with those of the employers. In Germany, there is financial support for this, as well as for technical aids such as electric wheelchairs, splints or adapted computer operating devices. Although this initially generates costs for both the public support system as well as for the employer, the productivity of individuals with SCI would make it worthwhile for the system as a whole. Financially, a re-integration would furthermore mean that the state can reduce or even discontinue financial support such as employment disability pension, thereby saving money. Here one should not expect any quick effects; after one year, the costs of a complex and costly rehabilitation are not yet balanced [27], signifying that long-term effects need to be considered. The importance of this investment becomes especially evident when calling to mind that 48.8% retire prematurely and when considering the relatively young age of many people with SCI. Also, interviewed persons are mostly well educated. Without professional re-integration, they would be missing as skilled workers in the labour market.

The financial situation is a double-edged issue: On the one hand, almost everyone in Germany who is unemployed can rely upon financial help, which is good and right for financial security reasons. Despite this, 22% of the respondents say that they do not want to work because they are afraid of losing their financial support. As a solution to this issue – next to informing those affected about the specific possibilities of support – a compromise between demands and support has to be found to relieve those affected of this concern and to motivate them to reintegrate into working life, with continuing financial assistance if necessary. Furthermore, it is crucial to demonstrate that those who return to work are financially better off than those who did not [28].

Also, support in finding a job, for example through case managers or a career advice centre, could help improving two of the factors that were given as reasons for being unemployed (“not finding a suitable job” (36%) and “not knowing how and where to look” (12%)). Vocational rehabilitation should ideally already be supported during in-patient rehabilitation, but at the latest must not be forgotten during permanent out-patient care, and should be continually re-addressed in the dialogue between doctors, patients, therapists and other professional groups in order not to miss any opportunities or wishes. It was also shown that it is very important for the prognosis of employment, whether patient and doctors agree on the further development. If patients do not agree with the results of the socio-medical assessment of performance by the team of therapists, the chance of returning to their workplace is lower [29].

It is known from inpatient rehabilitation for musculoskeletal diseases that 74% benefit from the therapy measures. However, the need for specific treatment pathways for further optimization became apparent [30]. These specific measures, depending on the functional impairments, must then be monitored scientifically, to determine the results, as is already the case with stroke, for example [31].

In the WHO documents and experts consensus papers, a strong consensus exists that for patients with SCI, in addition to timely and highly qualified first interventions, rehabilitation services must be provided for all patients. These services must be provided in the acute, post-acute, and long-term phase and should be delivered by well-trained health professionals working in multi-professional teams [32]. The ICF-classification could serve as a good basis for communication, as a common language between the professional groups to avoid misunderstandings [33]. In this context, it is essential to interview and involve the person concerned, as tensions between hope and expectations of work, as well as unaddressed needs for support, can create barriers to returning to work [34]. The importance of a new and diverse strategy for professional re-integration has already been emphasised in a meta-analysis [35]. However, this partly contrasts with the fact that less than half of those affected think about returning to work in rehabilitation immediately after the event that led to SCI [36]. The process of returning to social and community participation was identified as occurring in three main stages: withdrawal; re-emergence into society; and stability. Each stage consisted of adjustment and adaptation in several areas, including the loss of independence; the experience of being out in public; social networks; participation in productivity roles; and expectations regarding satisfactory social and community participation [37].

As we can see, the field of professional activity has many facets for people with SCI. While some are obvious, many are surprising concerning their importance for the person.

Because of the complexity of the described problem a study identified the need to investigate the policy development and education for all healthcare professionals supporting adults with SCI when undertaking to return to work to assist the transition further [38].

Three topics were identified in a scoping review that describes the meaning of work after SCI: Re-developing a sense of self, re-establishing place in the community and regaining economic self-sufficiency [39]. A survey of affected people in Sweden confirmed the desire of people with SCI to be treated as normally as possible, both in society and at work, and to be integrated as fully as possible into the team in all aspects [40]. Unfortunately, this is not always the case and discrimination also by colleagues has been reported as a problem [17]. As described in Article 23 of the United Nations Declaration of Human Rights, every adult has the right to gainful employment and adequate remuneration. The state has the responsibility to make this possible [41]. Not least, education for citizens without physical disabilities seems important in order to adequately promote and demand their tolerance for the needs of persons with funtional impairments.

The GerSCI project within the framework of the InSCI comparison is intended to contribute to this. The aim is to identify possible interrelationships, analyse probabilities and derive specific recommendations for politics and society. These recommendations should lead directly or indirectly to improve quality of life. The employment rate of people with disabilities, in the case of this survey, individuals with SCI has to come down. In order to achieve these goals, social medical experts, professional medical associations and interest groups, including those of affected persons, contribute to an effort to derive the most meaningful recommendations for action from the data obtained.

### Limitations

The response rate of 32.6% is not very high compared to other surveys. This could be due to the very extensive questionnaire. The questionnaire itself brings about possible selection bias because answering is a challenge for mental ability and motor activity.

The recruiting strategy also included possible selection bias because all involved persons were treated at least once in specialised SCI clinics. It is possible and even probable that the situation of persons not treated in such centres is even worse. Another limitation of the study was that only eight of a total of 27 specialised SCI centres in Germany participated. This could lead to regional ‘blind spots’, especially in the west and north of Germany.

## Conclusion and outlook

The persons with SCI in this survey are a professionally well-educated and for the most part, highly motivated group of people with most of them wanting to have gainful employment and considering themselves fit for work.

The data obtained allow identifying several factors which appear to influence employment. Of these, some cannot be changed, but many can be positively influenced by concerted efforts by society, politicians, employers and by the concerned people themselves. It is important to draw the right conclusions from the large data set. To that effect, our working group in Germany has already planned workshops between doctors, therapists, social workers and people with Spinal Cord Injuries in order to relate these data to everyday reality. Furthermore, meetings with German politicians have already taken place, and more are planned for the near future for the implementation of improvement plans derived from this project.

Entirely in line with the demands from the OECD report “Sickness, Disability and Work: Breaking the Barriers”: The best way to fight benefit dependence and exclusion among people with disability is to promote their re-integration into employment if they can and wish to work. Work: the best way to help people with health problems or disability [1].

## Data Availability

The original data are available on request to Mrs. Bökel, Hannover Medical School, Germany or at the InSCI Study Center at the Swiss Paraplegic Center, Nottwil, Switzerland.

## Abbreviations

GerSCI: German Spinal Cord Injury Survey
ICF: International Classification of Functioning, Disability and Health
Ilias: International Labour Market Integration Assessment in SCI
InSCI: International Spinal Cord Injury community survey
ISCoS: International Spinal Cord Society
ISPRM: International Society of Physical Rehabilitation and Medicine
MDS: Model Disability Survey
NEFI: Nottwil Environmental Factors Inventory
OECD: Organisation for Economic Co-operation and Development
OR: Odds ratio
SCI: Spinal Cord Injury
SCI-SCS: Spinal Cord Injury Secondary Comorbidity Scale
SD: Standard deviation
SGB: Social Code Book
SwiSCI: Swiss Spinal Cord Injury Community Survey
TSI: Time since injury
WHO: World Health Organization

## References

[1] Sickness, disability and work: breaking the barriers. 2010. OECD.

[2] BIH Bundesarbeitsgemeinschaft der Integrationsämter und Hauptfürsorgestellen. ZB Spezial Inklusionsvereinbarung.

[3] Aktion Mensch (ed.) Inklusionsbarometer 2019. https://www.aktion-mensch.de/inklusion/arbeit/inklusionsbarometer.html.

[4] Blumenthal M, Geng V, Egen C, Gutenbrunner C (2016). Querschnittlähmung in Deutschland – Forschungsdaten zur Gesundheit, Versorgungs- und Lebenssituation Betroffener. Phys Med Rehab Kuror.

[5] Giese R, Kaphengst C, Thietje R. Erfolg der beruflichen Wiedereingliederung bei querschnittgelähmten Rehabilitanden. In: Deutsche Rentenversicherung Bund, 21. Rehabilitationswissenschaftliches Kolloquium. DRV-Schriften Band 98. 2012.

[6] Gross-Hemmi MH, Post MWM, Ehrmann C, Fekete C, Hasnan N, Middleton JW, Reinhardt JD, Strøm V, Stucki G (2017). Study protocol of the International Spinal Cord Injury (InSCI) community survey. Am J Phys Med Rehabil.

[7] Fekete C, Brach M, Ehrmann C, Post MWM, Stucki G. Cohort profile of the International Spinal Cord Injury (InSCI) Community Survey implemented in 22 countries. Arch Phys Med Rehabil. 2020. 10.1016/j.apmr.2020.01.022 32533933

[8] Bökel A, Blumenthal M, Egen C, Geng V, Gutenbrunner C. Querschnittlähmung in Deutschland: Eine nationale Befragung (German Spinal Cord Injury Survey (GerSCI) Teilprojekt des Spinal Cord Injury Community Survey (InSCI))*. *doi 10.26068/mhhrpm/20191009-003.

[9] Bökel A, Egen C, Gutenbrunner C, Weidner N, Moosburger J, Abel F-R, Rupp R, Kalke Y-B, Liebscher T, Kurze I, Sauer M, Geng V, Sturm C. Querschnittlähmung in Deutschland – eine Befragung zur Lebens- und Versorgungssituation von Menschen mit Querschnittlähmung. Die Rehabilitation. 2020. 10.1055/a-1071-5935 31962349

[10] Sinden KE, Martin Ginis KA (2013). Identifying occupational attributes of jobs performed after spinal cord injury: implications for vocational rehabilitation. Int J Rehabil Res.

[11] Gross-Hemmi MH et al. Participation in people living with spinal cord injury in Switzerland: Degree and associated factors. 10.1016/j.apmr.2019.03.018 31026462

[12] Hess DW, Ripley DL, McKinley WO, Tewksbury M (2000). Predictors for return to work after spinal cord injury: A 3-year multicenter analysis. Arch Phys Med Rehabil.

[13] Reinhardt JD, Post MWM, Fekete C, Trezzini B, Brinkhof MWG (2016). Labor Market Integration of People with Disabilities: Results from the Swiss Spinal Cord Injury Cohort Study. PLoS ONE.

[14] Schwegler U, Nützi M, Marti A, Trezzini B. Pre- and post-injury job type distributions of individuals with SCI in relation to structural changes in the labor market: a comparative analysis based on findings from the Swiss Spinal Cord Injury Cohort Study. J Spinal Cord Med. 2019: 1–12. 10.1080/10790268.2019.1573346 PMC7919908 30714888

[15] Nützi M, Trezzini B, Ronca E, Schwegler U (2017). Key demands and characteristics of occupations performed by individuals with spinal cord injury living in Switzerland. Spinal cord.

[16] Nützi M, Trezzini B, Staubli S, Ronca E, Schwegler U. An interdisciplinary approach to job matching: developing an occupation-specific job matching tool for reintegrating persons with spinal cord injury into the labor market. Disabil Rehabil. 2019: 1–15. 10.1080/09638288.2018.1561958 30929524

[17] Lidal IB, Huynh TK, Biering-Sørensen F (2007). Return to work following spinal cord injury: a review. Disabil Rehabil.

[18] Huang I-C (2017). Employment outcomes following spinal cord injury in Taiwan. Int J Rehabil Res.

[19] Franceschini M, Pagliacci MC, Russo T, Felzani G, Aito S, Marini C (2012). Occurrence and predictors of employment after traumatic spinal cord injury: the GISEM Study. Spinal Cord.

[20] Schönherr MC, Groothoff JW, Mulder GA, Eisma WH (2005). Vocational perspectives after spinal cord injury. Clin Rehabil.

[21] Thomas FP, Goetz LL, Dixon T, Ho C, Holmes SA, Sandford P, Smith S, Ottomanelli L (2014). Optimizing medical care to facilitate and sustain employment after spinal cord injury. J Rehabil Res Dev.

[22] van Velzen JM, van Leeuwen CMC, de Groot S, van der Woude LHV, Faber WXM, Post MW (2012). Return to work five years after spinal cord injury inpatient rehabilitation: is it related to wheelchair capacity at discharge?. J Rehabil Med.

[23] Müller R, Brinkhof MWG, Arnet U, Hinrichs T, Landmann G, Jordan X, Béchir M (2017). Prevalence and associated factors of pain in the Swiss spinal cord injury population. Spinal cord.

[24] Krause JS, Dismuke-Greer CE, Reed KS, Li C (2020). Employment status, hours working, and gainful earnings after spinal cord injury: relationship with pain, prescription medications for pain, and nonprescription opioid use. Spinal cord.

[25] Siddall PJ, Middleton JW (2015). Spinal cord injury-induced pain: mechanisms and treatments. Pain management.

[26] Hug K, Hummel B, Rinaldo C, Saleh C, Lochmann H, Hund-Georgiadis M. Arbeitstätigkeit nach einer Rückenmarksläsion – Daten aus dem Ambulatorium einer paraplegiologischen Rehabilitationsklinik. Die Rehabilitation. 2019. 10.1055/a-0958-0264 31412400

[27] Sinnott PL, Joyce V, Su P, Ottomanelli L, Goetz LL, Wagner TH (2014). Cost-effectiveness of supported employment for veterans with spinal cord injuries. Arch Phys Med Rehabil.

[28] Ferdiana A, Post MWM, de Groot S, Bültmann U, van der Klink JJL (2014). Predictors of return to work 5 years after discharge for wheelchair-dependent individuals with spinal cord injury. J Rehabil Med.

[29] Kessemeier FM, Bassler M, Petermann F, Kobelt-Pönicke A (2019). Welche PatientInnen sind mit dem Ergebnis ihrer sozialmedizinischen Leistungsbeurteilung nicht einverstanden und welche Behandlung brauchen sie?. Phys Med Rehab Kuror.

[30] Grote V, Böttcher E, Mur E, Kullich W, Puff H (2019). Medizinische Ergebnisqualität: Unspezifische outcome-parameter einer stationären rehabilitation des Stütz- und Bewegungsapparates in Österreich. Phys Med Rehab Kuror.

[31] Knapp S, Oesterle U, Kaluscha R, Krischak G (2020). Evaluation eines Behandlungskonzeptes für die neurologische Anschlussrehabilitation nach Schlaganfall (Phase D): Studienprotokoll für eine Multicenterstudie. Phys Med Rehab Kuror.

[32] Gutenbrunner C, Blumenthal M, Geng V, Egen C (2017). Rehabilitation services provision and payment. Am J Phys Med Rehabil.

[33] Weichert H, Hennecke C, Keppeler R, Glaesener J-J (2020). Implementierung von ICF als Sprache und Struktur in die Teambesprechung der muskulo-skelettalen rehabilitation. Phys Med Rehab Kuror.

[34] Holmlund L, Guidetti S, Eriksson G, Asaba E (2018). Return to work in the context of everyday life 7–11 years after spinal cord injury - a follow-up study. Disabil Rehabil.

[35] Hilton G, Unsworth C, Murphy G (2018). The experience of attempting to return to work following spinal cord injury: a systematic review of the qualitative literature. Disabil Rehabil.

[36] Kennedy P, Hasson L (2016). Return-to-work intentions during spinal cord injury rehabilitation: an audit of employment outcomes. Spinal Cord.

[37] Barclay L, Lentin P, Bourke-Taylor H, McDonald R (2019). The experiences of social and community participation of people with non-traumatic spinal cord injury. Aust Occup Ther J.

[38] Vrbska V, Pratt AL. The experiences of active participation in returning to paid employment for adults with spinal cord injury in the United Kingdom. bura.brunel.ac.uk.

[39] Ullah MM, Fossey E, Stuckey R (2018). The meaning of work after spinal cord injury: a scoping review. Spinal Cord.

[40] Holmlund L, Hultling C, Asaba E (2018). Mapping out one’s own paths toward work: focus on experiences of return to work after spinal cord injury. Qual Health Res.

[41] United Nations. Universal Declaration of Human Rights (2017 [III] A).

